# Transdiagnostic neuroimaging markers of psychiatric risk: A narrative review

**DOI:** 10.1016/j.nicl.2021.102634

**Published:** 2021-03-17

**Authors:** Lucy D. Vanes, Raymond J. Dolan

**Affiliations:** a Centre for the Developing Brain, Department of Perinatal Imaging and Health, King’s College London, United Kingdom; b Max Planck UCL Centre for Computational Psychiatry and Ageing Research, University College London, London, United Kingdom

**Keywords:** General psychopathology, Transdiagnostic risk

## Abstract

Several decades of neuroimaging research in psychiatry have shed light on structural and functional neural abnormalities associated with individual psychiatric disorders. However, there is increasing evidence for substantial overlap in the patterns of neural dysfunction seen across disorders, suggesting that risk for psychiatric illness may be shared across diagnostic boundaries. Gaining insights on the existence of shared neural mechanisms which may transdiagnostically underlie psychopathology is important for psychiatric research in order to tease apart the unique and common aspects of different disorders, but also clinically, so as to help identify individuals early on who may be biologically vulnerable to psychiatric disorder in general. In this narrative review, we first evaluate recent studies investigating the functional and structural neural correlates of a general psychopathology factor, which is thought to reflect the shared variance across common mental health symptoms and therefore index psychiatric vulnerability. We then link insights from this research to existing *meta*-analytic evidence for shared patterns of neural dysfunction across categorical psychiatric disorders. We conclude by providing an integrative account of vulnerability to mental illness, whereby delayed or disrupted maturation of large-scale networks (particularly default-mode, executive, and sensorimotor networks), and more generally between-network connectivity, results in a compromised ability to integrate and switch between internally and externally focused tasks.

## Highlights

• We review the literature on neural correlates of a general psychopathology factor
• General psychopathology relates to structural and functional neurodevelopment
• Disrupted network connectivity maturation may underlie psychiatric vulnerability

## 1 Introduction

Advances in human neuroimaging have not only greatly improved our understanding of the neural underpinnings of perceptual and cognitive processes in the healthy brain, but also of the emergence of neuropsychiatric conditions when these processes go awry. Importantly, the neuroscientific perspective on key psychological functions has shifted from a dominant modular view to one that takes account of distributed networks, whereby concerted activity in multiple brain regions supports a range of cognitive and behavioural tasks (Fox et al., 2005, Power et al., 2011). Accordingly, mounting evidence for network-level dysfunction in many psychiatric disorders indicates that this level of analysis is crucial for developing novel interventions in psychiatry (Buckholtz and Meyer-Lindenberg, 2012, van den Heuvel and Sporns, 2019, Van Essen and Barch, 2015).

Progress in understanding network dysfunctions in psychiatric disorders raises a question as to how specific these dysfunctions are to individual conditions and to which extent patterns of dysconnectivity are shared across diagnostic boundaries. Magnetic resonance imaging (MRI) research in psychiatry has traditionally focused on single diagnoses, and distinct patterns of dysconnectivity have been observed for individual disorders including schizophrenia (Fitzsimmons et al., 2013), depression (Gudayol-Ferré et al., 2015), attention-deficit hyperactivity disorder (Konrad and Eickhoff, 2010), and obsessive compulsive disorder (OCD) (Piras et al., 2013). However, it is becoming increasingly clear that there is also considerable overlap across diagnoses in regions showing abnormal connectivity, measured both functionally (i.e., temporal correlations between spatially disparate brain regions) and structurally (i.e., integrity of connecting white matter tracts of the brain) (Goodkind et al., 2015, van den Heuvel and Sporns, 2019). For example, striatal dysconnectivity has frequently been implicated in the development of schizophrenia (Karcher et al., 2019), but so too is it thought to play a role in other disorders such as OCD (Fitzgerald et al., 2011), bipolar disorder (Teng et al., 2014), and depression (Furman et al., 2011). This raises the distinct possibility that brain connectivity measures could capture shared psychopathological risk across a range of disorders (Buckholtz and Meyer-Lindenberg, 2012).

The importance of a better understanding of the nature of these shared neural abnormalities across disorders is becoming increasingly clear. Not only will it help in teasing apart general (i.e., affecting risk for all mental illnesses) from specific (i.e., affecting risk for a particular disorder) pathophysiological processes that lead to psychiatric conditions, by extension improving our understanding of the unique aspects of individual disorders; it promises also to shed light on abnormal processes that render individuals vulnerable to a range of mental health problems. This type of general vulnerability plausibly operates at the circuit (i.e. network) level, given the intrinsic organisation of the brain into networks which coordinate the complex interplay of mental processes known to be affected in many psychiatric disorders, where key examples include cognitive control (McTeague et al., 2017) and emotional processing (McTeague et al., 2020).

It is now widely appreciated that the brain shows continuing maturation throughout adolescence and into early adulthood (Lebel and Beaulieu, 2011, Mills et al., 2016, Tamnes et al., 2013). Many psychiatric disorders are thought to have developmental origins, and this is underlined by the fact that a majority of individuals who experience a mental disorder have their first onset by age 18 (Caspi et al., 2020). The peak age of onset for anxiety disorders and impulse control disorders is in mid- to late childhood; psychotic and substance abuse disorders first occur most frequently during the transition from adolescence to adulthood, while mood disorders tend to emerge in early adulthood (Kessler et al., 2005, Meyer and Lee, 2019, Paus et al., 2008). As such, identifying reliable neural predictive markers of psychiatric vulnerability during adolescence is of particular importance. In recent years, there has been an increasing shift towards a dimensional approach to psychiatric nosology (Insel et al., 2010). This parallels an increasing recognition of substantial comorbidity in psychiatry (Kessler et al., 2012), bolstering the notion that risk for common psychiatric disorders is shared across diagnostic boundaries. A growing literature is now emerging that addresses the neural substrate of a transdiagnostic general psychopathology (‘*p*’) factor. This factor, described in more detail in the next section, captures shared variance across common mental health symptoms (Caspi et al., 2014, Lahey et al., 2012) and is therefore thought to reflect the extent of underlying vulnerability to (any) psychiatric disorder. This type of general psychopathology factor has been observed in a number of studies in healthy community samples of adolescents and young adults (Carragher et al., 2016, Laceulle et al., 2019, Patalay et al., 2015, St Clair et al., 2017). Within this framework, sub-clinical expression of psychopathology in the general population is considered temporally and phenomenologically continuous with clinical symptomatology (consistent with the notion of an “extended phenotype”). This in turn can help provide insight into mechanisms underlying general vulnerability to psychiatric symptoms preceding clinical illness onset.

An emerging body of work now seeks to identify possible candidate regions and circuits implicated in transdiagnostic psychiatric risk. Given the multitude of possible modalities and methods that can be used to address this issue, it is important to attempt a synthesis of key findings from this research so as to ascertain which mechanisms most reliably relate to vulnerability. In particular, important questions arise with respect to the spatial and temporal relationship between abnormal structure and function of the brain, which may shed light on the manner in which abnormalities – especially at the circuit level – give rise to behavioural and clinical manifestations of mental ill health. While investigating general psychopathology in young, representative cohort samples is critical in order to understand putatively developmental mechanisms leading up to vulnerability, there is an equally important need to link these findings to insights from clinical observations on individuals with diagnosed mental health disorders. This will ultimately help determine the meaningfulness of established neural markers and their implications for both prevention and treatment in psychiatry.

The aim of this narrative review is to examine critically the current evidence for transdiagnostic neural phenotypes of general psychopathology. First, we will summarise and evaluate recent studies investigating the functional and structural neural correlates of a general psychopathology factor in predominantly healthy developmental cohorts. In adopting a dimensional approach, these studies provide insights into potential neural markers of vulnerability to general psychopathology. In order to evaluate whether these markers are indeed reflective of clinically relevant psychiatric risk, we then link these insights to existing *meta*-analytic work investigating shared patterns of neural abnormality across categorical psychiatric disorders. We conclude by providing an integrative account of vulnerability to mental illness that focuses on neurodevelopmental abnormalities.

## 2 Transdiagnostic risk and the general psychopathology factor

The empirical phenomenon of a positive manifold amongst psychological symptoms has long been observed in psychiatry (Boyd et al., 1984). That is, an individual exhibiting increased symptomatology in one mental health domain is also likely to exhibit increased symptomatology in another, resulting in high rates of comorbidity amongst psychiatric disorders (Caspi et al., 2020, Kessler et al., 2012). A recent body of research has captured this phenomenon factor-analytically within the framework of a bifactor model (Holzinger and Swineford, 1937). Bifactor modelling is an increasingly popular quantitative method of capturing covariation of observed indicators (e.g. symptoms), resulting in a single “general” latent factor, on which all indicators load, in addition to specific (“group”) factors. Indicators may load on the general factor and only one group factor. Applied to psychopathology data collected across a number of symptom domains (usually self-report questionnaires), the general factor (also termed the general psychopathology or “p” factor) thus captures the shared variance amongst all individual symptom items, while the specific factors independently capture the unique variance of individual symptom domains (such as internalising, externalising, and thought disorder) (Caspi et al., 2014, Laceulle et al., 2015, Lahey et al., 2012). The *p*-factor is consequently thought to capture a dimensional overall burden of psychopathology and has found traction in recent research that has sought to characterise demographic, cognitive, genetic, and neurobiological correlates of psychopathology through early development and into adulthood (Allegrini et al., 2020, Deutz et al., 2020, Lahey et al., 2017, Martel et al., 2017, Murray et al., 2016, Zald and Lahey, 2017).

While there is strong support for diagnostically agnostic dimensional approaches to characterising the structure of psychopathology (Insel et al., 2010), the growing popularity of the bifactor model has also generated recent criticism (Bonifay et al., 2017, Bornovalova et al., 2020, van Bork et al., 2017). This is primarily due to the bifactor model’s potential to overfit data, resulting in parameter instability and a tendency for global fit indices to inappropriately favour the bifactor model over alternatives. Therefore, it is important to appreciate that the mere statistical presence of a general factor does not necessarily imply that a single causal structure for psychopathology actually exists. Furthermore, the interpretability and meaning of the *p*-factor necessarily depends on the specific (symptom) indicators included in the model (Bornovalova et al., 2020). Nevertheless, these limitations notwithstanding, a general psychopathology factor has been shown to be predictive of outcomes as diverse as academic performance (Lahey et al., 2015, Patalay et al., 2015), executive function (Martel et al., 2017), and a range of psychosocial outcomes (Laceulle et al., 2019), supporting its interpretation as a meaningful construct. In addition, the p-factor has been shown to be highly heritable (Allegrini et al., 2020, Franke, 2016, Neumann et al., 2016), in line with high genetic risk shared across psychiatric disorders as demonstrated in twin and family studies (Kendler et al., 2011). Intriguingly, genetic evidence also provides support for a polygenic *p*-factor of psychiatric disorder, representing a single genetic dimension of the psychiatric spectrum (Selzam et al., 2018). Importantly, unique or reliable associations of the *p*-factor with key biological substrates, such as brain structure and function, would further support its validity as well as provide insight into mechanisms conferring vulnerability for psychopathology in general (Bornovalova et al., 2020, Zald and Lahey, 2017).

In the following, we review the recent literature investigating neural correlates of a *p*-factor in the context of bifactor modelling, but also include studies that use alternative methodological approaches such as independent component analysis (ICA) or principal component analysis (PCA) where these yield comparable measures of transdiagnostic general psychopathology. For example, averaging across independent components of psychopathology derived by ICA, or extracting the first principle component in PCA, have been used as alternative approaches yielding a measure of general psychopathology conceptually similar to the p-factor derived using bifactor analysis (i.e., a dimensional score reflecting non-specific, general symptom load). All studies included in this review use magnetic resonance imaging (MRI) to investigate the association between brain structure (Table 1) or function (Table 2) and a behavioural factor representing general psychopathology in predominantly healthy community cohorts. The overarching aim is to synthesise evidence for a neural signature of a general liability for mental ill-health.

## 3 The dimensional approach: Neuroimaging studies of general psychopathology

### 3.1 Structural imaging studies

In one of the first studies to examine structural brain correlates of the *p*-factor, Snyder et al. (2017) focused on grey matter volume (GMV) in early development. Based on previous findings that several psychiatric disorders show overlapping patterns of reduced GMV in regions regulating executive control (e.g., prefrontal cortex) and affective processes (e.g., amygdala and anterior insula) (Shang et al., 2014, Wise et al., 2017), the authors concentrated their attention on prefrontal and limbic regions of interest (including frontal cortical regions, anterior cingulate cortex, insula, amygdala, and hippocampus). The authors found that increased expression of the *p*-factor in children aged 6 to 11 was associated with reduced GMV in prefrontal (including dorsal and ventrolateral prefrontal cortex and orbitofrontal cortex), but not limbic or paralimbic, regions. They concluded that prefrontal areas within an executive control network are implicated in general psychopathology during early development. This observation is consistent with behavioural research demonstrating that the *p*-factor is predictive of executive functioning in children (Martel et al., 2017) and adolescents (Castellanos-Ryan et al., 2016). Taken together, Synder et al.’s findings provided a first suggestions that morphology of prefrontal regions, known to play an important role in executive control (Niendam et al., 2012), may be an early marker of general liability to psychopathology.

However, there is some evidence for a shift throughout development from a regionally specific to a more global role of cortical volume as a marker for general psychopathology. Using data from the Philadelphia Neurodevelopmental Cohort (PNC) (Satterthwaite et al., 2016), a large quasi-epidemiological study of brain development in 8–22 year-old subjects, Kaczkurkin et al. (2019) identified 18 structural covariance networks and assessed their association with a general *p*-factor. Within these networks, brain structure (based on cortical thickness and applied to volumetric data) consistently co-varies across participants and is considered to reflect the intrinsic organisation of the cortex into networks of shared functional specialisation, which can potentially be influenced by common mechanisms. Expression of *p* was associated with reduced cortical volume in *all* networks (but was unrelated to cortical thickness), suggesting a global effect of cortical volume which confers risk for psychopathology in this predominantly adolescent age group. A contemporaneous study in the same (PNC) cohort took a similar data-driven approach to identify components of structural covariation based on morphometric features, but focused primarily on the cerebellum (Moberget et al., 2019). In this study, the authors showed that general psychopathology, here derived from principal component analysis, related to cerebellar morphology; specifically, increased psychopathology was associated with reduced volume of a cerebellar component encompassing bilateral lobule VI and crus I. Interestingly, these two cerebellar regions are functionally connected to the salience network (Habas et al., 2009), implicated in detection, integration, and filtering of relevant interoceptive information as well as recruitment of relevant networks to modulate behaviour. While cerebellar morphology performed better than either cortical thickness or subcortical volumetric measures in predicting general psychopathology (Moberget et al., 2019) within this adolescent cohort, this contrasts with findings from an older sample of adults (aged 45) from the Dunedin study, a population-representative birth cohort, where *p*-factor scores related instead to globally reduced neocortical thickness (Romer et al., 2020).

Alnæs et al. (2018) assessed psychopathology in combination with diffusion MRI metrics indexing white matter integrity in 8–22 year-olds from the PNC cohort. Structural integrity of myelinated white matter tracts underpins overall connectivity between disparate brain regions and, as a consequence, enables effective integration of diverse neural processes. As such, disruptions to white matter microstructure are of considerable interest in psychiatric research, in light of reporting of widespread deficits across many disorders (Fields, 2008). In their analysis, Alnaes et al. applied linked independent component analysis to eight diffusion imaging derived maps reflecting different microstructural properties of white matter tracts. Crucially, an independent component reflecting the contribution of crossing fibres at the intersection of uncinate fasciculus and inferior fronto-occipital fasciculus was most strongly linked to overall measures of both psychopathology and cognition – here, an average taken over the weights of individual clinical and cognitive components, respectively. The results suggest that frontotemporal connectivity might serve as a transdiagnostic brain phenotype for psychiatric disorder (Alnæs et al., 2018). In contrast, findings from a more recent study suggest that general psychopathology is linked to global white matter microstructure rather than individual tracts (Neumann et al., 2020).

In a series of analyses, Romer and colleagues combined diffusion and volumetric data to study the structural neural correlates of the *p*-factor (Romer et al., 2018, Romer et al., 2019). In a large sample of university students, these authors found that higher *p*-factor scores were related to significantly reduced fractional anisotropy (FA) uniquely in the pons, as well as reduced volume in regions of the occipital lobe (left lingual gyrus and right intracalcarine cortex) and posterior cerebellum (left lobule VIIb). With cerebellar afferents passing through the pontine white matter tracts, the findings are suggestive of structural disruption of cerebellar circuitry and, perhaps more surprisingly, visual cortex as markers of transdiagnostic psychiatric risk. In a subsequent study conducted on data from 45-year old subjects in the Dunedin Study, Romer et al. (2019) replicated findings of a negative correlation between *p*-factor scores and pontine FA as well as visual cortex GMV. In contrast, they failed to replicate an association between psychopathology and cerebellar morphology: neither left posterior cerebellar GMV, as identified in their own previous study (Romer et al., 2018), nor independent cerebellar components derived analogously to Moberget et al. (2019) significantly predicted *p*-factor scores.

Taken together, these findings at face value generate interesting possible interpretations. The replicated negative association between pontine FA and the *p*-factor upholds the notion that efficient communication within circuits connecting prefrontal cortex and neocerebellum via pontine white matter pathways, putatively subserving executive control, capture resilience against psychopathology. The absence of a local structural cerebellar effect in Romer et al.’s replication study (Romer et al., 2019) implies that local processing is of lesser importance compared to structural integrity of these pontine pathways, reflecting connectivity within the fronto-cerebellar network. Alternatively, the finding of an association between cerebellar morphology and p-factor in younger (Romer et al., 2018) but not older (Romer et al., 2019) adults might also reflect a dynamic role of the cerebellum throughout the lifespan, where contributions of cerebellar structure to psychopathology are greater at a younger age. (Vanes et al., 2019)Finally, a correlation between reduced visual association cortex volume and the *p*-factor might reflect less efficient integration of bottom-up sensory signals with top-down executive control processes associated with a greater burden of psychopathology.

A large diffusion imaging study has investigated an association between the *p*-factor and microstructural integrity throughout the white matter skeleton in young adults (Hinton et al., 2019). While controlling for specific second-order internalising and externalising factors, this study revealed a positive relationship between general psychopathology and FA in the body of the corpus callosum, indicating interhemispheric hyperconnectivity. Although this finding is perhaps unexpected in light of the more common notion of hypoconnectivity in psychiatric disorders, the authors argue that psychopathology may be associated with an accelerated maturational trajectory followed by a steeper subsequent decline in the corpus callosum (Menks et al., 2017). However, inconsistent with this result is a further, albeit smaller, diffusion study showing a *negative* association between FA in the genu and body of the corpus callosum and a general psychopathology factor derived from a principle component analysis, controlling for unresolved loss or trauma (Riem et al., 2019). Unresolved-disorganised attachment, in turn controlling for general psychopathology, was uniquely associated with reduced FA in the splenium of the corpus callosum. While it is hard to reconcile these findings linking general psychopathology to callosal microstructure integrity with opposing directions of association (Hinton et al., 2019, Riem et al., 2019), they nevertheless highlight many of the difficulties inherent in comparing studies from this newly emerging literature on transdiagnostic psychopathology risk. Substantial differences in sample size, age, derivation of the general psychopathology factor, and covariates included in the analysis are likely to account for observed inconsistencies. For example, the inclusion of unresolved loss or trauma as covariate in Riem et al. (2019) is an important distinction from other studies, in light of substantial evidence that childhood adversity is associated with significant disturbances of white matter microstructure, particularly in the corpus callosum (McCarthy-Jones et al., 2018). This could therefore account in part for the opposing findings in the corpus callosum observed in these two studies (Hinton et al., 2019, Riem et al., 2019). Furthermore, the neural correlates of general psychopathology may take a dynamic course over the lifespan, thereby affecting associations observed in different age ranges (see Table 1). Interpretations particularly in terms of developmental processes therefore await rigorous replication in longitudinal designs.

In previous research from our group, Vanes et al. (2020) sought to examine an association between white matter development and general psychopathology in a longitudinal design, where we assessed myelin-sensitive magnetisation transfer in white matter tracts of 293 adolescents and young adults in combination with a transdiagnostic *p*-factor (Vanes et al., 2020). Higher expression of *p* in this cohort was associated with reduced longitudinal myelin increase in the dorsal cingulum and uncinate fasciculus, suggesting that slowed myelin maturation in these limbic association fibres is a marker for an increased risk of psychopathology, both in adolescence and young adulthood. In contrast, there was no cross-sectional relationship between white matter myelin content and level of psychopathology, highlighting the importance of longitudinal designs in capturing key developmental effects predictive of psychiatric risk. This, to our knowledge, is the only extant study investigating how myelin maturation in white matter tracts specifically relates to the *p*-factor. A further study in the PNC cohort assessed grey/white matter contrast (GWC), thought to reflect intracortical myelin, in combination with psychopathology (Norbom et al., 2019). Here, the authors calculated a measure of mean psychopathology, defined as the average across seven independent components derived using ICA on symptom scores from a neuropsychiatric interview. Strikingly, mean psychopathology was associated with GWC in several cortical regions showing regional proximity to the white matter tracts identified in our study, most prominently insular and cingulate cortices, but also visual cortex and lateral posterior regions bilaterally. Taken together, these studies indicate a potential role for abnormal maturation both of intracortical and white matter myelin in conferring risk for psychopathology.

**Table 1:** Structural magnetic resonance imaging studies of general psychopathology.

Authors (year)	Sample	Age	N	Psychopathology measure	Modality	Neuroimaging measure of interest	Findings
Snyder et al. (2017)	Community sample	6–11	254	*p*-factor from bifactor model	T1	GMV	Increased *p* is associated with reduced prefrontal GMV. No association with limbic regions of interest was observed.
Alnaes et al. (2018)	PNC	8–22	883	general psychopathology score from ICA and PCA	DTI	FA, MD, RD, L1, mode on anisotropy, fibre orientations, connectivity density	Increased general psychopathology is associated with reduced fronto-temporal crossing fibres (including uncinate fasciculus and inferior longitudinal fasciculus)
Romer et al. (2018)	DNS	18–22	951 /1200	*p*-factor from bifactor model	DTI, T1	FA, GMV	Increased *p* is associated with decreased pontine FA and decreased cerebellar and occipital GMV.
Moberget et al. (2019)	PNC	8–23	1401	general psychopathology factor from PCA	T1	cerebellar independent components	General psychopathology is best predicted by a cerebellar morphological component which shows connectivity with the frontoparietal network.
Kaczkurkin et al. (2019)	PNC	8–22	1394	*p*-factor from bifactor model	T1	CT, GMV	Increased *p* is associated with globally reduced GMV, but no association with cortical thickness was observed.
Romer et al. (2019)	Dunedin	45	854 / 860	*p*-factor from bifactor model	DTI, T1	FA, GMV	Increased *p* is associated with decreased pontine FA and decreased visual association cortex GMV. No association was observed with cerebellar morphology.
Hinton et al. (2019)	TTS	23–31	410	*p*-factor from bifactor model	DTI	FA, RD, AD	Increased *p* is associated with increased FA in the corpus callosum body (controlling for internalising and externalising factors).
Riem et al. (2019)	EPISCA	12–20	63	general psychopathology factor from PCA	DTI	FA	Increased general psychopathology is associated with reduced FA in the corpus callosum genu and body (controlling for unresolved-disorganised attachment resulting from trauma).
Norbom et al. (2019)	PNC	8–22	1467	general psychopathology score across ICA components	T1	GWC	Mean psychopathology is associated with grey/white matter contrast in insula and cingulate cortices and left lateral posterior cortex.
Romer et al. (2020)	Dunedin	45	875	*p*-factor from bifactor model	T1	CT, CSA	Increased *p* is associated with reduced cortical thickness across the neocortex, and is unrelated to cortical surface area.
Neumann et al (2020)	GenR	6–10	3030	General psychopathology score from structural equation model	DTI	FA	General psychopathology is associated with reduced global white matter factor based on FA across all tracts.
Vanes et al. (2020)	NSPN	14–24	293	p-factor from bifactor model	MT	MT saturation	Increased *p* is associated with slower myelin maturation in the dorsal cingulum and uncinate fasciculus.

Abbreviations: PNC = Philadelphia Neurodevelopmental Cohort. DNS = Duke Neurogenetics Study. TTS = Tennessee Twin Study. EPISCA: Emotional Pathways’ Imaging Study in Clinical Adolescents. GenR = Generation R Study. NSPN = Neuroscience in Psychiatry Network. GMV = Grey matter volume. ICA = independent component analysis. PCA = Principal component analysis. DTI = Diffusion tensor imaging. FA = fractional anisotropy. MD = Mean diffusivity. RD = Radial diffusivity. L1 = principal diffusion tensor imaging eigen value. AD = Axial diffusivity. CT = Cortical thickness. GWC = Grey/white matter contrast. CSA = Cortical surface area. MT = Magnetisation transfer.

### 3.2 Functional imaging studies

There is an extensive existing literature documenting cognitive impairments accompanied by abnormal recruitment of relevant brain networks within individual psychiatric conditions (e.g. see Minzenberg et al., 2009, Chen et al., 2011). However, it is less clear to what extent there is regional overlap in aberrant brain activation across syndromes. Consequently, recent studies have aimed to relate expression of transdiagnostic general psychopathology to patterns of brain activity and connectivity, either at rest or during cognitive task performance.

In one of the few studies examining correlates of the *p*-factor using task-based fMRI, Shanmugan et al. (2016) assessed executive network activation during an n-back working memory task in adolescents who were part of the PNC cohort. Increased levels of general psychopathology were associated with reduced activation within the salience network, including frontal pole, anterior cingulate cortex, anterior insula, thalamus and precuneus. This finding appears to converge with observations of impaired executive function across disorders, adding to the notion that executive system disruption may serve as a transdiagnostic marker of psychiatric risk (McTeague et al., 2016).

Using the same working memory task data, alongside an emotion recognition task and resting-state data in the same cohort, Kaufmann et al. (2017) conducted a neural “fingerprinting” procedure, where brain connectivity patterns across runs were assessed with the aim to identify a distinctiveness in each subject’s connectome (defined as the average accuracy in identifying a particular subject’s connectivity profile based on the same subject’s connectivity profile on a different run). Connectomes became more distinctive with age (over a range from age 8 to 22), and the rate of this maturational process was related to overall psychopathology. More specifically, while connectome distinctiveness did not relate to psychopathology under the age of 14, from this age onwards the rate of maturation of connectome distinctiveness was delayed in a subgroup of individuals with markedly increased symptoms across all clinical domains (based on principle component analysis). This pattern was evident in the full brain network, default mode network (DMN), motor network and visual I network. Increasing connectome distinctiveness throughout adolescence, reflecting increased stability of connectivity patterns across tasks, may constitute a neurodevelopmental process of network tuning over time. This may continue until a point of maximal context coherence for each individual is attained, potentially allowing for efficient environmental adaptation or task switching (Kaufmann et al., 2017). The findings therefore provide initial evidence that this developmental process might be transdiagnostically impaired in psychiatric disorders.

Several further studies have exploited data from the PNC cohort to examine functional activity in the adolescent brain at rest in relation to clinical symptoms (Barber et al., 2019, Kaczkurkin et al., 2018, Xia et al., 2018). Using arterial spin labelled perfusion MRI, Kaczkurkin et al. (2018) assessed cerebral blood flow (CBF) and its association with the *p*-factor. Higher expression of *p* was associated with increased CBF in the dorsal and rostral ACC, as well as right postcentral gyrus, parahippocampal cortex and midbrain. Given the role of dorsal ACC in regulating emotional responses, through modulation of affective limbic circuits, this region was used as a seed in further connectivity analyses. General psychopathology was associated with reduced connectivity between dorsal ACC and bilateral caudate, in line with prior evidence implicating cortico-striatal circuits in the pathogenesis of several psychiatric disorders (Fitzgerald et al., 2011, Furman et al., 2011, Tu et al., 2012).

Striatal connectivity was studied more specifically by Barber et al. (2019), who used resting-state fMRI to characterise age-normative maturation of striatal connections. Here, the dorsal posterior insula emerged as a strong contributor to general psychopathology, whereby functional putamen-insula connections were developmentally accelerated with increased psychopathology. Normative developmental effects indicated a reduction in striatal connectivity with age (for the majority of connecting regions other than the cerebellum), suggesting that an acceleration of this process reflects premature reduction in functional connectivity between putamen and dorsal posterior insula, a region known to be involved in sensorimotor interoception. Interestingly, while putamen connections to subcortical regions (globus pallidus and thalamus) were also developmentally accelerated, intra-striatal connections tended to be delayed with increasing psychopathology.

In an alternative approach, Xia et al. (2018) used canonical correlation analysis to map psychopathology to whole-brain resting-state connectivity in the PNC cohort. Although this method does not yield one single dimensional score for general psychopathology, its strength lies in the derivation of individual symptom dimensions that are informed by neurobiology and whose common and distinct neural signatures can, in principle, be discerned. Here, the resulting individual symptom dimensions (mood, psychosis, fear, and externalising) were each associated with dissociable alterations in brain connectivity, but all four dimensions showed a shared pattern of abnormal within-network connectivity of the DMN and fronto-parietal network, as well as reduced segregation between the DMN and executive (fronto-parietal and salience) networks.

Additional evidence implicating the DMN in general psychopathology comes from a large study in preadolescent children (Sato et al., 2016), which used machine learning to estimate a network maturity index from fractional amplitude of low frequency fluctuations (fALFFs) in DMN nodes at rest. This maturity index was calculated as the difference between each child’s actual age and the age predicted from fALFFs of the DMN, thus reflecting each individual’s deviation from the expected DMN maturity for their age group (e.g, a younger predicted than actual age reflects maturational delay relative to age-matched peers). In line with expectations, Sato et al. observed that increased psychopathology is associated with delayed DMN maturation in this age group (Sato et al., 2016). This effect was evident only when considering DMN maturation index as a categorical variable, explicitly differentiating between delayed, typical and precociously developing groups, but did not hold when entered as a continuous variable, suggesting, at most, a subtle effect in this young age group.

Elliott et al. (2018) used multivariate distance matrix regression (MDMR) to identify brain regions whose whole-brain connectivity was most associated with *p*-factor expression in young adults. MDMR evaluates, at each voxel, whether subjects who show high similarity (i.e. small distance) in whole-brain connectivity of that voxel also show high similarity in a particular behavioural phenotype (here, p-factor) (for a more detailed description of MDMR see Shehzad et al., 2014). This yielded a pattern largely consistent with the canonical resting-state visual processing network, indicating that subjects with similar levels of general psychopathology show similar visual area connectivity. Specifically, connectivity of visual association cortex with somatosensory regions decreased with increasing *p*-factor scores, while connectivity with transmodal association regions increased with *p*. Further investigation revealed that hyperconnectivity of visual cortex was most evident for the DMN and fronto-parietal network, potentially reflective of more effortful integration of bottom-up sensory information with attentional demands and executive processes.

The potential importance of somatosensory-motor network connectivity in adults is further highlighted in a study by Kebets et al. (2019), who studied resting state data in a sample including individuals both with and without psychiatric diagnoses. Dysconnectivity of somatomotor networks with both subcortical and cortical executive networks was associated with a general psychopathology component derived using partial least squares, and this pattern of dysconnectivity was furthermore shared with cognitive dysfunction and impulsivity components. Importantly, this effect was evident across health and pathology, suggesting a role for somatomotor networks as “transdiagnostic hubs” conferring risk for psychopathology in general.

**Table 2:** Functional magnetic resonance imaging studies of general psychopathology.

Authors (year)	Sample	Age	N	Psychopathology measure	Modality	Neuroimaging measure of interest	Findings
Sato et al. (2016)	HCP	6–12	654	*p*-factor from bifactor model	rs-fMRI	fALFF	Increased *p* is associated with delayed maturation of the DMN.
Shanmugan et al. (2016)	PNC	8–21	1129	*p*-factor from bifactor model	fMRI (n-back)	BOLD signal	Increased *p* is associated with a disturbed pattern of executive system recruitment, including hypoactivation of frontal pole, ACC, anterior insula, thalamus, and precuneus.
Kaufmann et al. (2017)	PNC	8–22	797	general psychopathology factor from PCA	fMRI (n-back, emotion recognition), rs-fMRI	BOLD signal	Increased general psychopathology is associated with decreased connectome distinctiveness, and a decreased rate of maturation thereof.
Elliott et al. (2018)	DNS	18–22	605	*p*-factor from bifactor model	rs-fMRI	BOLD signal	Increased *p* is associated with decreased connectivity between visual and somatosensory regions, but hyperconnectivity between visual association cortex and DMN as well as heteromodal fronto-parietal network
Kaczkurkin et al. (2018)	PNC	11–23	1042	*p*-factor from bifactor model	ASL	CBF	Increased *p* is associated with increased CBF in ACC, postcentral gyrus, parahippocampal cortex and midbrain; and with reduced connectivity between dorsal ACC and bilateral caudate.
Xia et al. (2018)	PNC	8–22	1600	Shared patterns across four symptom dimensions based on CCA	rs-fMRI	BOLD signal	Within-network dysconnectivity of the DMN and fronto-parietal network, as well as reduced segregation between DMN and executive networks (salience and fronto-parietal), is shared across all symptom dimensions.
Kebets et al. (2019)	CNP	21–50	272	general psychopathology factor from PLS	rs-fMRI	BOLD signal	Increased general psychopathology component (and cognitive dysfunction and impulsivity components) is associated with somatomotor dysconnectivity.
Barber et al. (2019)	PNC/PING	8–23 / 9–21	926	general psychopathology factor from PCA	rs-fMRI	BOLD signal	Increased general psychopathology is associated with developmentally delayed intrastriatal functional connectivity; and with accelerated connectivity between the putamen and insula, globus pallidus, thalamus and anterior temporal pole as well as accelerated connectivity between caudate and mPFC/OFC.

Abbreviations: HCP = Human Connectome Project. PNC = Philadelphia Neurodevelopmental Cohort. DNS = Duke Neurogenetics Study. CNP = Consortium for Neuropsychiatric Phenomics. PING = Pediatric Imaging, Neurocognition, and Genetics. rs-fMRI = Resting-state functional magnetic resonance imaging. fALFF = Fractional amplitude of low-frequency fluctuations. DMN = Default mode network. BOLD = Blood oxygen level dependent. ACC = Anterior cingulate cortex. ASL = Arterial spin labelling. CBF = Cerebral blood flow. CCA = Canonical correlation analysis. PLS = Partial least squares. PCA = Principal component analysis.

### 3.3 Summary

The studies we review on the neural correlates of general psychopathology encompass a wide array of methodologies, constrained by a focus on a variety of regions and mechanisms of interest (though it needs to be acknowledged that a striking proportion of studies were conducted in the very same cohort (Alnæs et al., 2018, Barber et al., 2019, Kaczkurkin et al., 2018, Kaczkurkin et al., 2019, Kaufmann et al., 2017, Moberget et al., 2019, Norbom et al., 2019, Shanmugan et al., 2016, Xia et al., 2018)). However, despite the range of findings in this admittedly young literature, a number of converging patterns are beginning to emerge.

First, abnormalities in the maturational processes of several networks during development appear to play a pivotal role in conferring a vulnerability to psychopathology. Unfortunately, there is a notable dearth of longitudinal studies which would be ideally suited to identifying true developmental effects, and aberrations thereof. Nevertheless, within the literature several findings point towards a delay or slower rate of maturation both in structural and functional aspects of the connectome which is associated with an increased risk of experiencing adverse mental health outcomes (Barber et al., 2019, Kaufmann et al., 2017, Sato et al., 2016, Vanes et al., 2020).

Secondly, structural imaging studies focusing on white matter integrity or myelin content (albeit small in number) tend to report more focal effects compared to studies focusing on grey matter volume or cortical thickness (with the exception of one study showing global white matter microstructural effects (Neumann et al., 2020)). Both diffusion (Alnæs et al., 2018) and myelin-sensitive magnetisation transfer (Vanes et al., 2020) findings point towards structural abnormalities in frontotemporal fibre pathways that connect the limbic system, for example the uncinate fasciculus, as key candidates associated with general psychopathology. This is reflected further in abnormal intracortical myelin in adjacent insular and cingulate regions (Norbom et al., 2019). Callosal findings, in contrast, remain inconsistent (Hinton et al., 2019, Riem et al., 2019), potentially reflecting more complex interactions with known effects of early life adversity on the corpus callosum (McCrory et al., 2010), while morphological findings in the grey matter suggest an increasingly global effect throughout the cortex with development (Kaczkurkin et al., 2019, Romer et al., 2020, Snyder et al., 2017).

Third, fMRI studies bring more specificity in terms of functional networks implicated in general psychopathology, and several key networks emerge across modalities and methods: notably, the DMN, executive (salience and fronto-parietal) networks, and sensorimotor network. The DMN, also known as the resting-state network, deactivates during task engagement and is involved in self-referential processes such as introspection and autobiographical memory retrieval (Raichle, 2015, Whitfield-Gabrieli and Ford, 2012). Delayed maturation of the DMN in terms of within-network connectivity (Sato et al., 2016) and overall distinctiveness (Kaufmann et al., 2017) may result in reduced segregation between this and executive networks involved in strategic cognitive processing (Xia et al., 2018). Furthermore, reduced activation and connectivity is evident in core nodes of the salience network (e.g. insula (Barber et al., 2019, Shanmugan et al., 2016) and dorsal anterior cingulate (Kaczkurkin et al., 2018)), known to mediate dynamic interactions between other large-scale networks (Menon and Uddin, 2010). An ensuing inability to switch effectively between relevant circuits, in response to environmental demands, may represent a fundamental mechanism leading to vulnerability to mental health symptoms.

The emergence of sensorimotor networks in relation to general psychopathology is perhaps surprising, given that these are rarely the explicit focus of psychiatric neuroimaging. However the potential role of somatosensory-motor networks in psychopathology is increasingly being recognised (Martino et al., 2016, Sha et al., 2019, Velasques et al., 2011), with their importance for interoceptive predictive processes (Barrett and Simmons, 2015) and bodily awareness (Blanke, 2012) being of particular interest. Sensorimotor connectivity has been implicated in psychotic illness, where perceptual and motor dysfunction may be mediated by disintegration between cognitive networks and sensory, somatosensory and motor circuits (Kaufmann et al., 2015). In a recent investigation, using data-driven clustering of a community sample based on a wide range of behavioural and clinical assessments, intrinsic functional connectivity of the somatomotor network differentiated between functionally adaptive and maladaptive groups (Van Dam et al., 2017), further underlining its potential importance in well-being and general function.

Overall, existing work on the neural substrates of general psychopathology points towards a role for delayed maturation of limbic and default mode connectivity and more generally reduced between-network connectivity, potentially resulting in a compromised ability to integrate and switch between internally (somatosensory-motor networks, DMN) and externally (executive networks) focused tasks. These findings emphasise the importance of investigating the neural underpinnings of psychiatric vulnerability at the network level, consistent with the recognition that the intrinsic organisation of the human brain into functional networks critically underpins effective mental functioning (Fox et al., 2005).

Although more research will be needed in order to replicate and further substantiate the neural correlates of general psychopathology reviewed here, these findings point towards biological validity of the *p*-factor. While statistical concerns have been raised concerning the conceptual interpretability of the general factor (Bonifay et al., 2017), the existence of underlying neural correlates, particularly those reflecting abnormal maturational processes, lend support to the idea that the general psychopathology factor may indeed reflect an underlying vulnerability to a wide array of mental health symptoms. Moreover this is bolstered by the fact that similar patterns can be observed when deriving a general psychopathology factor by alternative dimension reduction techniques, suggesting that a shared liability to different mental health problems may be, at least in part, neurodevelopmentally rooted.

In addition, multimodal studies combining structural and functional neuroimaging analyses with respect to general psychopathology are still lacking. Brain function and structure are tightly interconnected (Yee et al., 2018), and while brain function is frequently characterised as being constrained by anatomy, structure and function of the brain are in fact likely to interact bidirectionally (Sui et al., 2014). For example, functional networks are thought to emerge as a result of the underlying structural connectivity (Grayson et al., 2014, Van den Heuvel and Sporns, 2013), but evidence also suggests that functional activation can induce structural neuroplasticity, for example via activity-dependent myelination (Fields, 2015). Furthermore, it is also possible for structural changes (e.g. due to aberrant neurodevelopment following perinatal brain injury) to be, to some extent, compensated by functional reorganisation (Froudist-Walsh et al., 2015). Further research investigating the temporal correspondence of structural and functional neural correlates of general psychopathology will therefore be crucial to shed further light on the dynamic processes underlying vulnerability.

## 4 The diagnostic approach: Neural dysfunction across psychiatric disorders

Traditionally, psychiatric neuroimaging studies overwhelmingly consist of case-control designs focusing on single conditions, or comparisons of two (often phenotypically related) diagnoses. However, with a growing acknowledgment in psychiatric research of common comorbidities (Caspi et al., 2020) as well as symptomatic overlap across conditions (in line with long-standing clinical observations), the number of investigations comparing two or more psychiatric disorders has increased in recent years (Chang et al., 2018, de Lange et al., 2019, Huang et al., 2020, Koshiyama et al., 2019). An important development is a growing number of transdiagnostic *meta*-analyses of neuroimaging findings. These offer a distinct advantage of being able to compare diagnoses which were not included in the same original investigations, and thus address the potential fallacy of attributing undue specificity to findings observed in single-diagnosis studies. Indeed a recent *meta*-analysis of fMRI studies, over a broad range of psychiatric disorders, suggests little to no diagnostic specificity in abnormal task-related neural activation in patients compared to healthy controls (Sprooten et al., 2017). In line with this, increasing evidence points towards neurobiological commonalities across disorders that may constitute vulnerability “hot spots” that are shared across diagnostic categories.

In an influential *meta*-analysis, Goodkind et al. (2015) compared structural imaging studies across major axis I disorders, observing a shared pattern of reduced grey matter volume across disorders in dorsal anterior cingulate cortex (dACC) and bilateral anterior insula; putative hub regions anchoring the salience network. They showed also that, in the healthy brain, lower grey matter volume in these regions was systematically related to poorer performance on a measure of higher-level cognitive control, but not processing speed. Cognitive control deficits are observed in many psychiatric disorders and have been proposed as a transdiagnostic behavioural vulnerability factor for mental ill health (McTeague et al., 2016). Thus, in a subsequent *meta*-analysis investigating whether functional abnormalities exist paralleling the observed structural deficits across disorders, McTeague et al. (2017) included studies comparing functional activation during cognitive control tasks between patients with psychiatric diagnoses and healthy controls. A striking convergence with structural deficits was observed, in that patients showed abnormal activation in regions corresponding to interfacing regions of the salience and fronto-parietal networks, which are thought to coordinate their activity during tasks involving executive control as part of a broader “multiple-demand network”. Reduced activation was observed transdiagnostically in a dACC region corresponding to that prone to grey matter loss (Goodkind et al., 2015), in the right anterior insula, as well as in several lateral prefrontal and parietal regions. Interestingly, strong anterior insula dysfunction was already evident in child and adolescent psychiatric samples, suggesting an important role for this region in cognitive dyscontrol throughout development (McTeague et al., 2017).

The respective roles of the anterior insula and dACC in a so-called multiple demand system have been examined in a host of imaging studies. Within the salience network, the anterior insula is responsible for detecting and evaluating salient information with respect to its goal-relevance (Downar et al., 2002), forwarding signals for further cognitive processing (Menon and Uddin, 2010), and coordinating switching between default mode and fronto-parietal networks (Menon and Uddin, 2010, Sridharan et al., 2008). The dACC, in contrast, monitors performance and selects and modulates relevant motor responses in concert with motor networks (Botvinick et al., 2004, Downar et al., 2002, Shenhav et al., 2013). Indeed graph-analytical work supports the idea that dACC and insula interact in two distinct but adjacent anatomical circuits, subserving task control and salience processing, respectively (Power et al., 2011).

The notion that efficient integration of, and switching between, relevant networks is a key process in many psychiatric disorders is emphasised further in *meta*-analytic work by Sha et al. (2018). These authors showed that regions commonly affected across conditions, in terms of their functional connectivity, tended to be network connectors – nodes involved in inter-network communication and multiple cognitive functions – consistent with anatomical evidence that hubs of the connectome are disproportionately affected across (neurological and psychiatric) brain disorders (Crossley et al., 2016). This emphasises once again that while individual neural abnormalities – both on the functional and structural level – may be observed in regionally specific areas (e.g., anterior insula or dACC), these abnormalities can have profound implications at the network level.

The manner in which between-network connectivity is disrupted across disorders is not straightforward. A *meta*-analysis focusing on resting-state connectivity of the DMN, fronto-parietal network, and salience network found patterns of both hypo- and hyperconnectivity between these networks that were shared across eight psychiatric disorders (Sha et al., 2019). While hypoconnectivity between the DMN and salience network (in particular ventral insula and dACC), as well as between the fronto-parietal network and salience network, was evident across diagnoses, the DMN was also hyperconnected with components of the salience network (dorsal insula) and fronto-parietal network. This underlines the importance of distinct parts of the insula within the salience network, which display differing connectivity patterns likely responsible for distinct aspects of cognitive processing (Cauda et al., 2011). The same *meta*-analysis also demonstrated a shared pattern of grey matter reductions in regions similar to those reported by Goodkind et al. (2015), including prefrontal cortex, bilateral insula, and dACC. Regions showing abnormal functional connectivity were furthermore systematically related to both patterns of grey matter loss across disorders and general cognitive performance in healthy subjects.

These comprehensive *meta*-analyses of morphometric and functional imaging studies, including a wide array of psychiatric conditions, begin to map out a pattern of aberrant brain mechanisms shared across diagnostic boundaries. These include grey matter loss in key nodes of the salience network coupled with imbalances in connectivity within, and between, networks that support contextual shifting and inhibition of behavioural responses, largely converging with findings of *meta*-analyses focusing on narrower diagnostic ranges, such as anxiety and mood disorders (Janiri et al., 2020, Shang et al., 2014, Wise et al., 2017).

Less strong evidence exists with respect to white matter microstructural abnormalities shared across disorders. A recent study identified dissociable clusters of psychiatric patients displaying distinct patterns of abnormal white matter microstructure; interestingly, these clusters were not diagnostically specific, each having a similar distribution of patients with depressive, bipolar, psychotic, or anxiety related diagnoses (Hermens et al., 2019). In contrast, a clustering analysis of published effect sizes of regional patient-control differences in FA, derived from diagnosis-specific *meta*-analyses of several brain disorders, suggests that schizophrenia-spectrum disorders cluster with depressive and bipolar disorders, while post-traumatic stress and obsessive compulsive disorder cluster with traumatic brain injury (Kochunov et al., 2020). A *meta*-analysis of DTI studies in affective and anxiety disorders (Jenkins et al., 2016) suggests that these conditions are characterised by shared patterns of frontotemporal and frontoparietal white matter dysconnectivity, largely in the left superior longitudinal fasciculus (connecting aspects of the DMN with the fronto-parietal network), inferior fronto-occipital fasciculus (forming intra and cross-network connections of the salience and fronto-parietal networks) and uncinate fasciculus (connecting prefrontal cortex to limbic structures of the salience network). These tracts have also been implicated in psychotic disorders (Szeszko et al., 2008), though to our knowledge a comprehensive transdiagnostic *meta*-analysis of diffusion imaging studies does not exist.

## 5 Synthesis and conclusion

In this review, we first aimed to examine evidence for neurobiological correlates of transdiagnostic general psychopathology, thought to reflect a latent liability for mental illness. Existing research suggests that we can indeed delineate certain neural mechanisms associated with a general ‘*p*’-factor of psychopathology in community cohorts. We next assessed *meta*-analytic work on a large body of research studying the neural correlates of individual psychiatric disorders, which collectively points towards the fact that conditions that are traditionally diagnostically distinct share neural abnormalities particularly in brain networks that support flexible responding to changing environmental demands. Importantly, the emerging neuroimaging research on general psychopathology is beginning to suggest how these common abnormalities might develop. The evidence does not appear to indicate that any single clear-cut neural dysfunction accounts for general psychopathology during development. Rather, an ensemble of aberrant developmental patterns, impacting brain structure and function, are likely to conspire over time in mediating risk for psychopathology. A possible chronological account of these developmental patterns leading to vulnerability, based on the available neuroimaging evidence reviewed here, is described in the following.

During childhood, lower prefrontal grey matter volume may represent an early marker of susceptibility (Snyder et al., 2017). Our own research implicates a slowed maturation rate of myelinated white matter tracts connecting the frontal cortex to temporo-limbic areas during adolescence (Vanes et al., 2020), also reflected in aberrant microstructural patterns observed in these regions that are linked to greater symptom load (Alnæs et al., 2018). It is plausible that a disruption of inputs to cortical regions via these pathways places constraints on the development of stable, and flexibly interconnected, functional networks (Sato et al., 2016). This developmental tuning of network connectivity may be particularly important so as to enable adaptation to the unique demands of an individual’s environment, reflected in a distinctiveness for each individual’s connectome (Kaufmann et al., 2017). Over time, insufficient network connectivity may result in increasingly global effects on morphology throughout the cortical grey matter (Kaczkurkin et al., 2019, Romer et al., 2020, Romer et al., 2018), further compounding inefficient communication between disparate brain regions. The resulting dysconnectivity within and between key cognitive networks are likely to ultimately form the basis for vulnerability to psychiatric disorder.

Attempts to map the findings on general psychopathology in community cohorts onto the clinical neuroimaging literature are still lacking. To bridge the gap between these lines of investigation, it will be necessary not only to include more clinical cases in studies of the *p*-factor, but also to conduct longitudinal studies which track the neural correlates of general psychopathology throughout development and assess their predictive value for clinical outcomes as well as their correspondence to the neural correlates of symptoms at a clinical illness stage directly.

Our review of the *meta*-analytic evidence for transdiagnostic neural abnormalities in clinical samples suggests that there is some correspondence between dysfunctions seen across psychiatric disorders and those associated with general psychopathology. Specifically, many of the observed disruptions appear to converge on key nodes of the salience network, with the level of dysfunction scaling with psychopathology. The anterior insula is of particular interest here given its involvement in a multitude of cognitive processes implicated in mental illness, due to its central role in mediating between default mode and higher order cognitive networks. Its importance in generic salience monitoring can provide a parsimonious account for transdiagnostic influences, where a similar neurobiological abnormality might plausibly give rise to a range of different clinical manifestations (Menon, 2011), characterised by an inability to use externally or internally salient events to guide behaviour. These insights are important not only in the pursuit of identifying transdiagnostic markers of psychiatric risk, but also in the study of individual disorders and specific syndromes. Given the accumulating evidence for the existence of transdiagnostic neural correlates of general psychopathology, future research wishing to identify diagnostically specific markers of psychiatric illness may consider controlling for the effects of general psychopathology. This way, brain research will increasingly be able to tease apart general from specific aspects of mental illness, thus improving our understanding of possible pathways to different forms of psychopathology.

In order to address this question further, future research will need to go beyond simply identifying regions and circuits associated with general psychopathology. Identifying the locus of abnormality in the brain leaves many question unanswered. For example, there remains a chasm between these types of observations and the deeper question of how such deficits explain the phenomenon under investigation. Here, a richer connection to complementary disciplines such as computational neuroscience and preclinical research with animal models is needed, which can help clarify the nature of the transition from general vulnerability to clinically manifest dysfunction. The computational mechanisms by which regional abnormalities give rise to behavioural deficits and symptoms that straddle multiple psychiatric conditions require particular attention. Findings here have considerable potential to inform the development of novel interventions that can tackle psychiatric vulnerability early on. A continued multidisciplinary approach, including a significant increase of longitudinal and multimodal elements, will therefore be crucial in further elucidating the developmental course of neural mechanisms conferring risk for psychopathology.
